# Aberrant histone modifications in pediatric brain tumors

**DOI:** 10.3389/fonc.2025.1587157

**Published:** 2025-06-10

**Authors:** Erin T. Hamanishi, Derek Dang, Sriram Venneti

**Affiliations:** ^1^ Division of Pediatric Hematology, Oncology and Bone Marrow Transplant, Department of Pediatrics, University of Michigan Medical School, Ann Arbor, MI, United States; ^2^ Department of Pediatrics, University of Michigan Medical School, Ann Arbor, MI, United States; ^3^ Laboratory of Brain Tumor Metabolism and Epigenetics, Department of Pathology, University of Michigan Medical School, Ann Arbor, MI, United States; ^4^ Chad Carr Pediatric Brain Tumor Center, University of Michigan, Ann Arbor, MI, United States; ^5^ Department of Pathology, University of Michigan Medical School, Ann Arbor, MI, United States

**Keywords:** epigenetics, histone modifications, histone methylation, histone acetylation, pediatric

## Abstract

Epigenetic modifications, particularly histone post-translational modifications (PTMs), are central to pediatric brain tumor pathogenesis, impacting chromatin structure, gene expression, and genomic stability. Disruptions in histone PTMs, especially lysine methylation and acetylation, arising due to histone mutations or aberrant enzyme modulation are critical drivers of oncogenesis. Lysine methylation, catalyzed by histone methyltransferases (KMTs), modulates chromatin interactions and gene expression through activation or repression, depending on the methylation state and the specific histone residue. Key enzymes, including histone methyltransferases and demethylases, and associated proteins exemplify the functions of writers, readers, and erasers in maintaining histone modification balance. Similarly, histone acetylation, a dynamic process regulated by histone acetyltransferases (HATs) and histone deacetylases (HDACs), plays a crucial role in pediatric brain tumors. Alterations in these components lead to aberrant gene expression and tumorigenesis. Understanding these disrupted processes offers potential for targeted therapies to rewire oncogenic chromatin states and potentially improve patient outcomes.

## 1 Introduction

Pediatric brain cancers are the most common solid tumor in children, leading to significant morbidity and mortality (1). In contrast to many adult tumors, pediatric cancers are often characterized by a paucity of recurrent mutations and bear a relatively lower tumor mutational burden (2–4). In contrast, extensive research into pediatric brain tumors has revealed fundamental deregulation in chromatin biology and epigenomic alterations. These differences highlight the unique molecular landscape of pediatric malignancies, offering insights into how chromatin structure and gene regulation are disrupted in these cancers.

**Figure 1:**
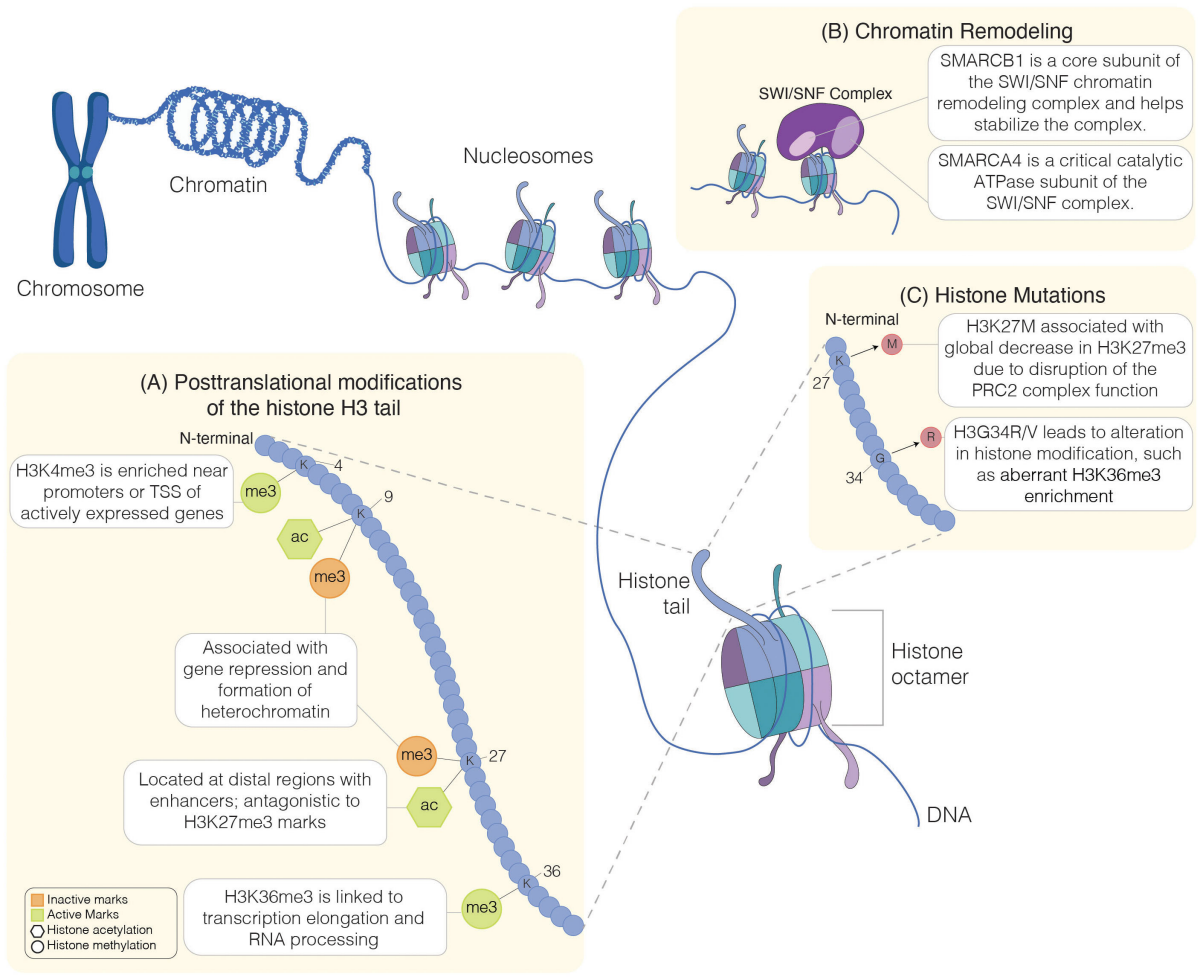
Regulation of chromatin structure. Schematic representation of chromatin organization. Chromatin is made up of DNA wound around histone proteins. Each histone complex comprises an octamer of histone protein, including H2A, H2B, H3, and H4 (7, 8). Chromatin remodeling, histone mutations, and histone modifications influence chromatin structure. Lysine-specific post-translational modifications of histone H3 tail **(A)** include methylation (me) and acetylation (ac). Histone modification can be inactive (orange symbols) or active (green symbols). Chromatin accessibility can be influenced by chromatin remodelers, such as the SWI/SNF complexes, which are composed of multiple subunits, such as the SMARCB1 subunit involved in the complex stabilization (9) or the catalytic subunit SMARCA4 (10) **(B)**. Histone mutations **(C)**, including H3K27M or H3G34R, interfere with histone modifications, leading to aberrant chromatin structure and accessibility. Chromosome, chromatin: Created in BioRender. Chung, C. (2025) https://BioRender.com/a87r173.

Within eukaryotic cells, DNA is organized into chromatin, a complex of DNA and proteins arranged into repeating units called nucleosomes. Each nucleosome consists of a core octamer of histone proteins around which approximately 146 base pairs of DNA are tightly wrapped, forming the fundamental unit of chromatin structure (5, 6). The core octamer comprises four core histones: H2A, H2B, H3, and H4 (7, 8) (**Figure 1**). Linker DNA connects these nucleosomes, and linker histone H1 binds to the nucleosome core at the sites where DNA enters and exits, contributing to chromatin structure and stability (11). Chromatin organization is dynamic and tightly regulated; modulation of chromatin structural changes plays a crucial role in regulating gene expression and maintaining genomic stability, underscoring the importance of chromatin’s dynamic nature in cellular function.

Disruption of chromatin regulation and structure can have profound effects, as alterations in chromatin due to mutations in histone post-translational modifications (PTMs) (**Figure 1A**), chromatin remodeling complexes (**Figure 1B**), histone proteins (**Figure 1C**), or DNA methylation can significantly impact gene expression. Such disruptions may lead to the aberrant activation of oncogenes or the silencing of tumor suppressor genes, both critical in cancer development (12). In the setting of cancer, sequencing efforts have revealed mutations in genes involved in chromatin organization and regulation (13, 14). Beyond cancer, disruptions in chromatin regulation are linked to various other diseases, including neurodegenerative disorders and developmental syndromes (15–17). These alterations can also impact normal cellular processes, such as DNA repair, replication, and cell differentiation, highlighting the essential role of chromatin in maintaining cellular and genomic integrity. Herein, we will focus on the impact of aberrant histone modifications underlying pediatric brain tumors. Understanding these disruptions provides crucial insights into disease mechanisms and identifies potential therapeutic targets for correcting these aberrant chromatin states.

## 2 Post-translational histone modifications

Histone PTMs, like other mechanisms of chromatin modifications, create changes in chromatin structure that can impact genomic stability and gene expression (18, 19). Diverse arrays of PTMs have been reported, with some of the most well-studied histone modifications reported on lysine residues in the N-terminal tail of H3 and H4 (20). The covalent modifications to histone residues consist primarily of methylation, acetylation, phosphorylation, and ubiquitination, although not limited to those mentioned. In the common paradigm of histone PTMs, distinct categories of proteins—referred to as “writers,” “readers,” and “erasers”—play crucial roles in regulating PTMs (21). Writers are enzymes that add specific PTMs to histones, such as methylation, acetylation, or phosphorylation. For instance, methyltransferases such as SET1 (histone-lysine N-methyltransferase, H3 lysine-4 specific - Saccharomyces cerevisiae) and EZH2 (enhancer of zeste 2) add methyl groups to lysine residues on histone tails (22–25). Similarly, histone acetyltransferases (HATs) can add acetyl groups to histone lysine residues (26–30). Readers are proteins that recognize and bind to these modifications, translating the histone code into functional outcomes by regulating gene expression. These include chromodomain-containing proteins like HP1 (Heterochromatin protein 1), which binds to methylated lysine residues (31, 32). Erasers are enzymes that remove or reverse these modifications. These include histone deacetylases (HDACs) or demethylases like KDM1A/LSD1 (33, 34). Together, these readers, writers, and erasers maintain the complex dynamic balance of histone modifications, essential for regulating gene expression, maintaining genomic stability, and coordinating cellular processes. Understanding how these chromatin-modifying processes are disrupted in pediatric brain tumors offers potential therapeutic targets for precision treatments to restore normal chromatin function and improve patient outcomes.

The methylation of basic residues, such as lysine, arginine, and histidine, is a well-studied process, and the methylation of lysine residues, specifically on histone H3 and H4, has been extensively reviewed (35). Lysine can be mono- (me1), di- (me2), or trimethylated (me3), whereas arginine can be mono- or di-methylated, for example. Unlike the function of other PTMs, histone methylation does not affect the charge of the histone residue; instead, it regulates interaction with chromatin-binding proteins, thus impacting gene expression. Histone methylation at lysine residues can be associated with either transcriptional activation or repression, depending on the methylation level or the lysine residue involved (35, 36). Generally, H3K4 or H3K36 methylation is associated with the activation of genes. For example, H3K4 methylation marks are enriched near transcription start sites (TSS), and H3K4me2/me3 are strongly associated with euchromatin formation and active transcription. Certain transcriptional coactivators and chromatin remodeling complexes, such as the SAGA complex, recognize this modification, leading to the recruitment of RNA polymerase II and gene transcription (37, 38). Global increase in H3K4me3 positively correlates with higher grade and poor survival in certain pediatric brain tumors, such as ependymomas (39) and pediatric high-grade gliomas (pHGG) (40). H3K36me3 marks are typically localized to bodies of active genes and are associated with transcription elongation and RNA processing (41). In G34-mutant diffuse hemispheric gliomas (DHG), H3K36me3 marks are decreased in cis; however, they have preserved global levels (42). On the other hand, trimethylation of H3K9 (H3K9me3) or H3K27 (H3K27me3) is associated with repression of gene expression (36). A decrease in global H3K27me3 is commonly noted in certain high-grade tumors, such as pediatric high-grade gliomas, and the dysregulation of H3K27me3 can contribute to the oncogenesis of these tumors (43, 44). In addition to histone methylation, acetylation of lysine residues in the histone tail influences chromatin structure and gene expression, generally activating transcription. Along with the global loss of H3K27me3 marks, there is a reciprocal gain of H3K27ac across the genome in pHGG (45). Together, alterations in histone modifications can drive tumorigenesis through aberrant regulation of chromatin structure and gene expression (**Figure 1A**).

Histone methylation occurs via the writer proteins known as histone methyltransferases. Lysine methyltransferases (KMTs) catalyze the methylation of lysine residues by donating a methyl group from S-Adenosyl-L-Methionine (SAM). Histone methylation was first reported in the 1960s. However, researchers began to pay significant attention to the role of histone methyltransferases in the early 2000s with the discovery of SUV39H1 (KMT1) and EZH2 (46–49). These enzymes contain SET (Su(var)3–9, enhancer of zeste, and Trithorax) domains (SET-domain protein methyltransferase family) that play critical roles in methylation of histone lysine residues (50). SUV39H1 (KMT1) and its homolog SUV39H2 (KMT1B) are involved in H3K9 methylation (H3K9me2/me3) (51). EZH2 mediates trimethylation of H3K27 (H3K27me3) and is a key component of the polycomb repressive complex 2 (PRC2) (52). These enzymes can facilitate a repressive chromatin state by silencing gene expression by H3K9me3 and H3K27me3 enrichment. The first histone demethylase, KDM1A/LSD, was described in 2004, which removes the methyl mark from H3K4 (33). Since then, an extensive family of KMTs has been characterized; many principal writers (KMTs) and erasers (KDMs) have been identified, often for specific residues (34). Together, the combined functions of KMTs and KDMs can orchestrate complex patterns of genomic methylation and demethylation to regulate multiple cellular functions.

## 3 Pediatric brain tumors

**Figure 2:**
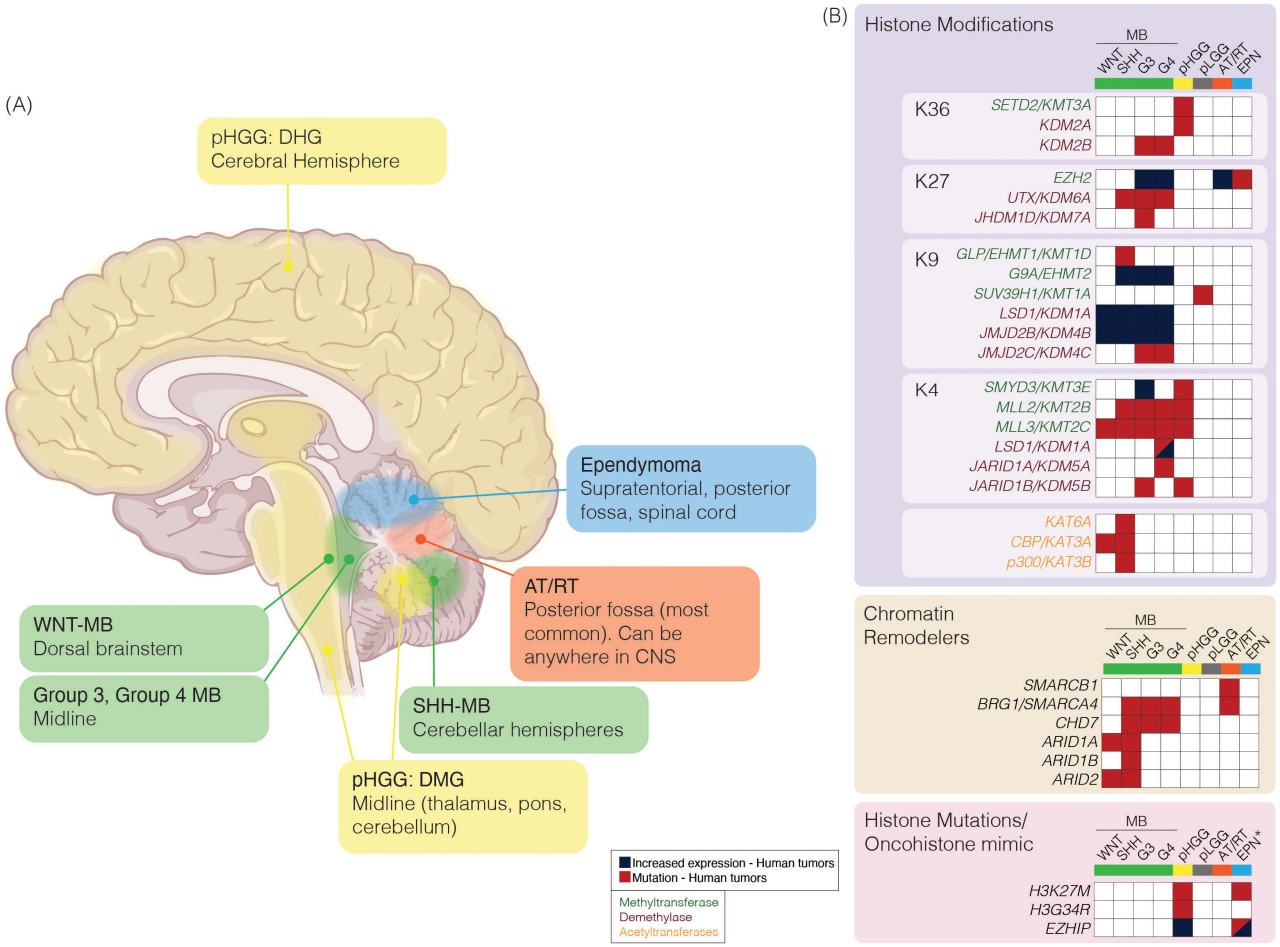
**(A)** Schematic of pediatric brain tumor location and characteristic epigenetic modifications. Pediatric brain tumors vary in anatomic location (left) and alterations in expression or mutations in epigenetic regulators (right). Medulloblastoma (MB; green) can be divided into subgroups based on clinical and molecular characteristics. Pediatric high-grade gliomas (pHGG; yellow) can be further characterized by characteristic histone mutations (pink box); diffuse hemispheric gliomas (DHG) are associated with H3G34R/V mutation, or diffuse midline glioma (DMG) are associated with H3K27M mutation. Atypical teratoid/rhabdoid tumor (AT/RT; orange) are most commonly located in the posterior fossa; however, it can be found anywhere in the CNS among pediatric patients. Ependymoma (EPN; blue) can be found in three anatomic compartments, supratentorial (ST), posterior fossa (PF) or spinal (SP). **(B)** Epigenetic alterations reported in at least one tumor sample from previously reported molecular characterization efforts in MB (53–59), pHGG (60–64), pediatric low-grade glioma (pLGG; grey) (65) or AT/RT (66–70). Mutations or alterations in expression among writers [histone methyltransferases (green text) or histone acetyltransferases (orange text)], erasers [histone demethylases (purple text)], chromatin remodelers, or histone mutations/oncohistone mimics are represented. *Increased expression or mutations in EZHIP reported, or H3K27M histone mutations reported specifically in PFA-EPN. Brain: Created in BioRender. https://BioRender.com/tmiyitf.

Pediatric brain tumors encompass a diverse spectrum of malignancies, each defined by distinct biological and clinical characteristics (**Figures 2A, B**). They most commonly arise in the hindbrain/posterior fossa (PF) region. This region of the brain houses critical structures, including the brainstem and cerebellum. Medulloblastomas (MB) are the most common brain tumor in children (1) and arise from the cerebellum. They are classified into four distinct subgroups: WNT, SHH, Group 3, and Group 4 (71, 72). Each subgroup is characterized by unique driver genetic events, proposed cell-of-origin, and distinct clinical implications (53, 73, 74), and are now further subdivided into several subtypes (54, 55, 75). Despite exhibiting a relative paucity of mutations across MB, alterations in epigenetic regulators and gene expression contribute to the heterogeneity and pathogenesis of MB.

Other aggressive brain tumors arising in children and adolescents include high-grade gliomas, ependymomas, and atypical teratoid/rhabdoid tumors (AT/RT). High-grade gliomas are characterized by infiltrative growth and malignant behavior. Mutations leading to the amino acid substitutions at lysine 27 to methionine (collectively referred to as H3K27M) in the N-terminal tail of histone H3 were identified in *H3-3A* (previously called *H3F3A*) and *H3-C2* (previously called *HIST1H3B*) (60, 76) (**Figure 2B**). Additionally, *H3-3A* mutations at glycine 34 to either arginine or valine (referred to as H3G34R/V) were noted and were mutually exclusive from H3K27M mutations (60). *H3-3A* encodes the non-canonical variant H3.3, while *H3-C2* encodes canonical, cell cycle-couple H3.1 (77–79). Subsequent studies have shown rare H3K27M tumors involving *H3-C3* (also encodes H3.1) (80, 81).

Ependymomas (EPN) exhibit variable biological behavior depending on their location within the central nervous system (82). Classification of EPN has further subdivided this disease entity into subgroups based on anatomical region and molecular characteristics (83). A majority of posterior fossa EPN tumors fall within the PF-A subgroup and are characterized by global hypomethylation of H3K27 (84–86). AT/RTs are highly aggressive and often present in very young children. They are driven by loss-of-function *SMARCB1* gene alterations (66). SMARCB1 is a critical component of the chromatin remodeler SWI/SNF (SWItch/Sucrose Non-Fermentable) complex. The SWI/SNF complex regulates nucleosome incorporation and the eviction of genes and functions in tandem with histone PTM modifiers (87, 88). Rare loss-of-function mutations in *SMARCA4*, another SWI/SNF component, have been reported in AT/RT (67, 68). A comprehensive understanding of the epigenetic alterations in these tumors is essential for dissecting the mechanisms underpinning their pathogenesis and developing effective and targeted therapies (**Figure 2B**).

## 4 Post-translational histone modifications in the developing hindbrain

Pediatric brain tumors most commonly arise within the hindbrain/PF region. Histone modifications are key to brain development, as illustrated in the development of the hindbrain and cerebellum. Understanding this complex phenomenon may help understand how these pathways are deregulated in pediatric brain cancers. The cerebellum is a highly foliated structure composed of many different types of neurons and astrocytes. Accordingly, the development of the human cerebellum is a highly complex process that begins one month after conception and extends into the second postnatal year (89, 90). Many efforts have been made to establish the genetic and developmental programs that underlie cerebellar ontogenesis. These efforts led to the identification of two primary zones of neurogenesis in the developing cerebellum: the ventricular zone and the rhombic lip (91). The cerebellar ventricular zone gives rise to GABAergic cerebellar neuronal derivatives, including Purkinje cells, GABAergic cerebellar nuclei, Bergmann glial cells, and inhibitory interneurons (91–94). On the other hand, cerebellar glutamatergic derivatives, including granule neuron progenitors, glutamatergic cerebellar nuclei, and unipolar brush cells, arise from the rhombic lip (95). Although human cerebellar development research continues to be obfuscated by the lack of relevant tissues and the intrinsic discordance between human and murine cerebellar development (90), there is growing evidence that supports critical roles for epigenetics in the proper development of this vital hindbrain structure.

The expression of proliferation and differentiation genes is tightly regulated by epigenetic modifications during mammalian cerebellar development. This is particularly well-documented in granule neurons, the most common neuron in the brain (96). Granule neuron progenitor (GNPs) cells are born in the rhombic lip and migrate along the cerebellar anlage, where they eventually form the cerebellar granular layer. Bivalent chromatin are regions of DNA that are simultaneously marked by both activating H3K4me3 and repressive H3K27me3 (97). This dual enrichment of both repressive and activation marks keeps genes in a “poised” state that can be readily modulated. Bivalent chromatin is particularly important in embryonic stem cells, as it helps maintain their undifferentiated state by keeping developmental genes poised for activation when necessary (97). Bivalent chromatin plays an important role during the proliferative phase of nascent GNPs, and these cells maintain high levels of H3K4me3 at genes associated with cell cycling and suppress differentiation genes via increased H3K27me3 (98). Accordingly, knockout of *Ezh2* in the developing mouse cerebellum decreases GNP and Purkinje cell proliferation (99). Histone modifications of genes that encode key effector proteins in signaling pathways can also affect GNP proliferation. The Sonic hedgehog (SHH) pathway is a key driver of granule neuron proliferation (100). SHH directly induces HDAC1, which in turn deacetylates gene regulatory regions of Gli2 and allows Gli2 to translocate to the nucleus and induce Gli1 expression (101). Gli1 promotes genes related to cell cycling and proliferation (101). Both HAT (Gcn5) and other HDAC (HDAC2, HDAC3) enzymes can also act directly on cell cycling gene programs to ensure proper GNP proliferation (102–104). ATP-dependent chromatin remodeler complexes, including Chd7 (99, 105), Snf2h (106), and npBAF (107), increase chromatin accessibility of pro-proliferation genes and regulate expression of genes that induce differentiation, such as reelin (105). Improper proliferation of GNPs caused by mutations in chromatin-remodeling enzymes causes cerebellar hypoplasia, underlying the importance of these complexes in the maintenance of adequate GNP pools (99, 105–107).

As GNPs stop proliferating and begin to differentiate into mature granule neurons, they exhibit simultaneous increase of H3K9me3 and H3K27me3 at cell cycling and proliferation genes and activating marks H3K4me3, H3K9ac, H3K14ac, H3K27ac at genes essential for synaptogenesis, ion channels, and cell adhesion (98, 108–111). Whereas HDAC1 activity induced GNP proliferation, the inhibition of HDAC1 via activation of pro-neurotrophin receptor p75NTR induces cell cycle arrest and subsequent neutrophin activity. These processes contribute to shifting cells towards differentiation (112). DNA methylation is also dynamically regulated during granule neuron differentiation. TET demethylases are highly expressed during this time and increase 5-hydroxymethylcytosine (5hmC) at genes associated with axonal guidance and ion channels (113). 5hmc is also present in differentiated Purkinje cells, suggesting an important role for this epigenetic mark among multiple cellular niches (114, 115). Concurrently, 5-methylcytosine (5mc) is increased at genes that promote proliferation and cell cycling (98). Chromatin-remodeling enzymes can also impact GNP differentiation. As GNPs mature, the nucleosome remodeling and deacetylase (NuRD) complex silences genes expressed in immature GNPs that induce ectopic early synaptogenesis, while its CHD4 subunit increases transcription of genes essential for dendrite pruning by inducing active chromatin (116). To summarize, histone tail modifying enzymes, DNA methylation modulators, and chromatin-remodeler complexes work in synchrony to modulate gene expression throughout cerebellar development which allows for proper GNP proliferation and differentiation.

## 5 Deregulated histone modifications in pediatric brain tumors

### 5.1 Oncohistones and oncohistone-like proteins

Recurrent somatic histone mutations in childhood brain tumors were discovered in 2012 through exome sequencing of pediatric glial tumors, including H3K27M and H3G34R/V mutations (**Figure 1C**) (60, 76). H3K27M and H3G34R/V can arise in differing age groups and within distinct anatomic regions of the brain. H3K27M gliomas arise mainly in younger children from the midline of the CNS and are collectively designated diffuse midline gliomas (DMGs) (71). These midline structures include the pons [where they are referred to as diffuse intrinsic pontine gliomas (DIPG)], thalamus, cerebellum, and spine (71). In contrast, H3G34R/V mutations are hemispheric, frequently observed in older children and adolescents, and are termed DHG H3-G34-mutant (71). These mutant histones, referred to as oncohistones, are associated with distinct gene expression profiles and global DNA methylation patterns (117).

H3K27M mutations occur most frequently in the non-canonical H3.3 (approximately 60-70%), whereas mutations targeting H3.1 or H3.2 are identified at a lower frequency in DMGs (118–120). H3.1 and H3.2 K27M tumors are located primarily in the brainstem, restricted to the pons, and often associated with younger age. In contrast, those harboring an H3.3 K27M are found across the midline, often in older children and adults (119, 121). In addition to the hallmark mutation, H3K27M, many DMGs harbor additional genetic alterations. The genetic mutations associated with H3.3 and H3.1/H3.2 vary. For example, H3.1 and H3.2 K27M DMGs are associated with activin A receptor type I (*ACVR1*) mutations. The gain of function mutations in *ACVR1* leads to the hyperactivation of the *bone morphogenic protein* (*BMP*) signaling pathway (122). Meanwhile, H3.3 mutant variants are commonly linked with loss of function alterations in platelet-derived growth factor receptor A (*PDGFRA*), ultimately leading to activation of downstream PI3K/AKT/mTOR and Ras/Raf/MEK/ERK downstream signaling (63, 121–125).

H3K27M mutations cause global H3K27me3 reduction due to dominant negative effects on the PRC2 complex (43, 126, 127). Once PRC2 is recruited to specific sites in the genome, it spreads and deposits H3K27me3 in adjacent regions (128–130). H3K27M can bind to EZH2/PRC2 and inhibit this spreading function of PRC2, resulting in a global reduction in H3K27me3 levels (43, 131–133). Despite this global decrease in H3K27me3, these high-affinity PRC2 genomic loci retain H3K27me3 in these tumors (127, 131, 134–137). H3K27M mutations can reprogram other histone PTMs including H3K27ac (discussed below), H3K36me3, H3K4me3 and bivalent histone marks (138–148).

EZHIP protein (EZH Inhibitory Protein or Cxorf67 or Catacomb) is an oncohistone-like protein expressed in the testis and important for spermatogenesis (149). EZHIP is overexpressed in majority of childhood posterior fossa group-A (PFA) ependymomas, resulting in a global reduction of H3K27me3 levels (150). EZHIP contains a methionine residue at position 406 that enables EZHIP to phenotypically mimics H3K27M by binding to and inhibiting the function of EZH2/PRC2 complex (126, 131, 134, 135, 146, 149, 151, 152). H3K27ac is an activating mark that opposes H3K27me3 and localizes to gene promoters and enhancers. Global H3K27me3 reduction in H3K27M gliomas and PFA ependymomas is associated with a global increase in H3K27ac levels (153). Genomic distribution of H3K27ac in these tumors converges on several neuro-developmental related enhancers and super-enhancers contributing to aberrant differentiation tumor cell states (45, 133, 154–165). Rare population of H3-wildtype, low-H3K27me3 DMGs overexpress EZHIP, and similarly small percentage of PFAs harbor H3K27M mutations (64, 71). Because DMGs can harbor both H3K27M mutations and overexpress EZHIP in rare tumors, they are designated collectively as DMG, H3-K27-altered (71).

Like H3K27 mutant tumors, DHG H3-G34-mutant tumors are characterized by a *H3-3A* mutation targeting the N-terminal tail of the histone protein. However, unlike H3K27M, the H3G34 mutation leads to glycine replacement by arginine or valine (60, 121). H3G34 mutant tumors are characteristically located in the cerebral hemispheres, with a propensity for the temporoparietal hemispheres. These tumors are most prevalent in adolescents and young adults (12–35 years) (166). Khazaei et al. demonstrated that H3-G34 substitutions lead to distinct phenotypic outcomes affecting neurodevelopment by altering the epigenome (167). Specifically, H3-G34R mutations reduce H3K36me2 and H3K36me3 levels on the mutant histone tail, disrupting the recruitment and distribution of DNMT3A deposition and mCH methylation (167). In general, when compared to other pHGG, H3-G43-mutant tumors are hypomethylated (117, 139). Many H3-G34 mutant tumors also bear activating mutations in *PDGFRA* or abnormal PDGFRA activation through enhancer hijacking (168). Abnormal downstream signaling of PDGFRA increases cell survival and proliferation, promoting gliomagenesis (168, 169). They are also often associated with loss of function mutations in *TP53 and ATRX* (encoding a chromatin remodeling protein) (117, 121). In a genetically engineered mouse model, *Atrx* loss in the presence of H3.3G34R upregulates of HOX genes and inhibits differentiation pathways (170). The replacement of glycine by arginine or valine leads to decreased SET Domain Containing 2, Histone Lysine Methyltransferase (SETD2) activity, ultimately resulting in altered PTMs of nearby H3K36 and H3K27 (42, 171–175). These epigenetic state-driven gene expression profiles map to interneuron-like GABAergic states and can impact the tumor microenvironment (176). To gain deeper insight into the immunological landscape of H3-G34-altered tumors, Garcia-Fabiani et al. examined the epigenetic and transcriptomic reprogramming in H3.3-G34R tumors. They found that the H3-G34R mutation reshapes the tumor microenvironment, leading to upregulation of immune-related genes and activation of the JAK/STAT pathway (177).

Given the need to further characterize the histone mutational landscape in cancers, comprehensive sequencing efforts have identified histone mutations in various cancers, including mutations in both the tail and the globular domains (178–180). Analysis of cancer genomes across various age groups, including adults, adolescents, and young adults, reveals that approximately 11% carry mutations in histone-encoding genes (180). Among CNS tumors, there is an increased prevalence of histone mutations among pediatric, adolescent, and young adults (AYA) when compared to adults. Not surprisingly, many of these histone mutations identified in the pediatric and AYA population were H3 K27/G34 mutations; however, non-H3, core, and linker mutations were also identified in a variety of CNS malignancies, including atypical teratoid/rhabdoid tumor (ATRT), DMG, HGG, ependymoma, and MB (180). Although mutant oncohistones may be a hallmark of tumorigenesis, it is evident through extensive investigations that they do not act alone but instead in concert with other genetic alterations, leading to the oncogenesis of these brain tumors.

### 5.2 Aberrant histone modifications in pediatric brain tumors

#### 5.2.1 Histone methylation

The activity and balance of specific KMTs and KDMs regulate the genomic enrichment of both repressive and activating histone marks. Disruption in the expression or function of these enzymes has been implicated in the pathogenesis of pediatric brain tumors. This is particularly evident in MB across many of the molecular subgroups. Molecular studies have identified numerous mutations that alter histone methylation patterns. Key alterations among KMTs in pediatric brain tumors include aberrant expression of EZH2, NSD1, SETD1A, SMYD3, MLL2 (KMT2D), and G9A (EHMT2) (40, 181–184). Conversely, mutations in KDMs are also frequently observed, for example amplification of JMJD family proteins such as JMJD2C (KDM4C), JMJD2B (KDM4B), and JMJD3 (KDM6B), as well as mutations in MYST3 and UTX (KDM6A) (181–184). In MB, mutations in KDM6A and MLL2 are associated with a loss of H3K27me3 (Dubuc et al., 2013). Additionally, global hypomethylation of H3K9 has been reported in approximately 40% of MB cases compared to normal brain tissue, and *in vitro* restoration of genes regulating H3K9 methylation results in a decrease in cell proliferation (54). Together, epigenetic dysregulation in pediatric brain cancers through altered histone methylation patterns plays a crucial role in the pathogenesis of these devastating tumors.

##### 5.2.1.1 Histone methyltransferases

The most studied methyltransferase in pediatric brain tumors is EZH2, a critical component of PRC2. PRC2 is involved in differentiation, proliferation, and maintenance of cellular identity in pediatric brain cancers, including H3K27- altered DMG, PFA ependymomas, MB, and AT/RT (185). Despite the global reduction of H3K27me3, H3K27-altered DMGs and PFA ependymomas retain genomic H3K27me3 to repress gene expression at high-affinity PRC2 sites, including the CDKN2A/B locus encoding the senesce-associated protein p16. Inhibition of EZH2 in these tumors is therapeutic by lowering H3K27me3 at these sites, leading to increased gene expression, including p16 (132, 135, 186–189).

PRC2 is composed of four core subunits: the catalytic EZH2 (responsible for methylation of lysine 27), EED, SUZ12, and RBAp46/48, in addition to several additional axillary subunits (190). Recruitment of PRC2 through EED to H3K27me3 stimulates the catalytic activity of EZH2 (190). Complete loss of *Eed* acts as a tumor suppressor in murine MB models, whereas mosaic *Eed* mutations can promote tumor growth, highlighting the impact of PRC2 heterogeneity driving tumor growth in MB (191). Deletion of *Eed* destabilizes PRC2 (192), and deletion of *Eed* or *Ezh2* in SHH-MB models is sufficient to promote the expression of genes typically suppressed by PRC2, specifically promoting myeloid differentiation and tumor progression (192).

MB tumors demonstrate elevated levels of EZH2 across groups, with the highest levels appreciated in groups 3 and 4 (56, 193). Subsequent alterations in genomic H3K27me3 can contribute to stem-like tumor cell states in groups 3 and 4 MB (191). EZH2 interacts with maternal embryonic leucine-zipper kinase (MELK), a member of the AMPK protein kinase family involved in the regulation of cell cycle and cellular function (194). Along with EZH2, MELK is frequently upregulated in MB and associated with reduced survival (195). MELK binds to and phosphorylates EZH2, thus working together to promote cancer stem-like cell proliferation and stemness (195).

Therapeutic inhibition of EZH2 in SHH-MB cells promotes tumor cell differentiation, impairs tumor growth and proliferation, and reduces stemness, suggesting that EZH2 represents a promising druggable target, which shows significantly reduced proliferation and impaired self-renewal in response to EZH2 inhibition (196–198). Human and mouse MB cells from the SHH-MB subgroup significantly reduced proliferation and impaired self-renewal in response to EZH2 inhibition (196, 197). Similarly, treatment with EZH2 inhibitors extended survival in SHH and Group 3 MB xenograft models (198). This suggests that EZH2 inhibition has promising evidence supporting reduced MB growth in a subset of MB *in vitro* and *in vivo*. However, inhibition of EZH2 in MB must be approached cautiously, as inhibition of EZH2 can lead to *Gfi1* upregulation through epigenetic remodeling, promoting tumor progression in MYC-driven Group 3 MB (196, 199), supporting a nuanced role for EZH2 in MB.

In cancers, including MB, microRNAs (miRNAs) are implicated in cancer initiation and progression through their crucial role in regulating gene expression (200). MiRNAs can act as tumor suppressors or oncomiR. In MB, miRNA profiling identified several miRNAs with unique profiles, with only a few upregulated and the majority downregulated in MB (201–203). The relationship between miRNAs and the KMT, EZH2, is complex. For example, during development, miR-10 downregulates key midbrain markers, such as *Otx2*, and upregulates hindbrain markers, such as *Gbx2* (204). In many cancer types, including some subgroups of MB, miR-10 family members are dysregulated (203, 205) leading to altered expression of their downstream targets. In group 3 MB, *Otx2* is frequently overexpressed (206). When OTX2 is silenced in MB tumorsopheres, EZH2 and SUZ12 levels decreased, suggesting that OTX2 plays a role in the regulation of PRC2 (207). Alternatively, EZH2 can be regulated by specific miRNAs, such as miR-101-3p and miR-423-5p, which have been identified of negative regulators of EZH2 in MB (208). Another example is the exosomal miR-130b-3p, which functions as a tumor suppressor *in vitro* and *in vivo* in MB (209). Specifically, EZH2 was identified as a target gene of miR-101-3p in MB (208). In the absence of EZH2, the inhibitory effect of the exosomal miR-130b-3p is lost, suggesting that miR-130b-3p mediates MB progression and may be a potential therapeutic target for the treatment of pediatric MB.

In addition to MB, aberrant EZH2 expression has been reported in AT/RT (210). The absence of SMARCB1 protein in AT/RT promotes *EZH2* expression (210, 211). Disruption of EZH2 via genetic or pharmacological inhibitors impairs cell growth, self-renewal and may potently sensitize ATRT cells to radiation therapy (69, 210). AT/RT tumors can also show a global increase in H3K37me3, independent of EZH2 expression (69). Although global increase in H3K27m3 is observed in ATRT, specific genomic regions were identified with higher H3K27ac occupancy in association with BET bromodomain-containing protein 4 (BET4) (212). *In vitro* and *in vivo*, combination therapy with EZH2 and BET4 inhibitors reduced cell proliferation and invasiveness (213). Interestingly, in SMARCB1-deficient AT/RT tumors, pharmacological inhibition of EZH2 induces the viral mimicry response (214).

Tazemetostat (EPZ-6438) is a potent and selective EZH2 inhibitor. Initial preclinical studies demonstrated that it induced apoptosis and differentiation in SMARCB1-deleted rhabdoid tumors *in vitro* and *in vivo* (215), with significant anti-tumor activity observed in rhabdoid tumor models, including AT/RT, but variable responses across other pediatric solid tumors (216). Subsequent preclinical studies demonstrated therapeutic potential in variety of pediatric CNS tumors, such as MB with wild type p53, AT/RT, or HGG, supporting further evaluation in pediatric brain tumors with EZH2 overexpression, though combination strategies may be needed to overcome resistance and intratumoral heterogeneity (198, 217). Encouraging preclinical evidence led to a phase 1 clinical trial (NCT02601937) examining tazemetostat monotherapy in pediatric patients with relapsed or refractory SMARCB1 (also known as INI1) negative tumors, such as malignant rhabdoid tumors (MRT), including AT/RT, or other SMARCB1-deficient tumors and synovial sarcoma. Among subjects with AT/RT, one had a complete response, and 5/21 had an objective response, with a 6.5-month duration of response (218). Additionally, in the phase 2 NCI-COG pediatric MATCH trial (NCT03213665), Arm C, tazemetostat was evaluated in a variety of pediatric tumors harboring EZH2 mutation or SMARCB1 or SMARCA4 loss (219). Within this trial, two pediatric CNS malignancies were represented, including AT/RT (n=8) and ependymoma (n=1), among other tumors, including MRT, epithelioid sarcoma, renal medullary carcinoma, hepatocellular carcinoma, Ewing sarcoma, and Langerhans cell histiocytosis (219). Although treatment with tazemetostat in this trial did not meet its primary efficacy endpoint, a diverse array of tumor diagnoses and molecular alterations were represented (219). Vejmelkova et al. reported the results of a small cohort of four pediatric patients with primary AT/RT treated with tazemetostat maintenance after the completion of upfront therapy with surgery, radiotherapy (older than 2 years), and chemotherapy. The most significant adverse effects were thrombocytopenia or other cytopenias requiring dose reduction (220). However, resistance to tazemetostat in patient-derived SMARCB1-deficient epithelioid sarcomas or rhabdoid tumors has been observed. Multiple factors can contribute to the development of resistance to EZH2 inhibitors. NSD1 is a histone H3 lysine 36 methyltransferase and was identified by CRISPR screens as a critical regulator of resistance to EZH2 inhibitors in AT/RT and extra-CNS rhabdoid tumors (221). Additionally, functional sequencing has uncovered distinct acquired mutations affecting the RB1/E2F axis that decouple EZH2-dependent differentiation from cell-cycle control, thus circumventing the drug’s intended mechanism (222). Beyond the intricacies of EZH2 inhibition, the broader landscape of histone modification offers additional avenues for therapeutic intervention.

G9a (euchromatic histone lysine methyltransferase 2, EHMT2) is another nuclear KMT belonging to the Su(var) 3–9 family that catalyzes histone H3K9 methylation (223, 224). G9a is higher in a subset of MB tumors, specifically those belonging to groups 3 and 4 (57). In conjunction with G9a, the repressor element-1 silencing transcription factor (REST) works to repress transcription by modifying chromatin through methylation of H3K9. In MB, increased expression of REST and, in turn, G9a activity leads to the subsequent downregulation of USP37 through methylation at its promoter region. USP37 is a component of the ubiquitin system implicated in the regulation of cell proliferation and reduced expression (225). Higher expression of G9a and REST both may be indicators of poor prognosis in this subset of MB (57, 225). Interestingly, higher levels of REST and USP7 (deubiquitylase) are reported in SHH-MB and are associated with increased LSD1/KDM1A expression (226). *In vitro*, cell migration was promoted by REST elevation in conjunction with elevated LSD1, and pharmacological inhibition of LSD1 decreased cell migration and viability (226). While REST is elevated in multiple MB subtypes, the distinct co-expression of opposing histone modifying enzymes like G9a and LSD1/KDM1A reveals that REST’s functional consequences are dependent on the specific epigenetic context, underscoring the complexity of its role in MB.

##### 5.2.1.2 Histone demethylases

Like KMTs, the role of KDMs has been extensively studied in the context of cancer (227, 228). More specifically, in MB, multiple KDMs have been implicated with aberrant methylation patterns of H3K27 and H3K4 (56, 184). KDM1A, also known as lysine-specific histone demethylase 1A (LSD1), is a H3K4 specific KDM. It is overexpressed across all subgroups of MB (229). Lsd1/Kdm1a knockdown was associated with apoptosis and suppression of proliferation; similarly, using a KDM1A inhibitor, NCL-1, inhibited the growth of MB cells (229). Inhibition of LSD1 sensitized H3K27M gliomas to HDAC inhibitors, promoted differentiation pathways, and induced natural killer (NK) cell infiltration into the tumor microenvironment (148, 230). KDM6 subfamily members are involved in the demethylation of di- and trimethylated H3K27, and dysregulation of KDM6s plays an important role in various cancers (231). In MB, KDM6 plays a role in oncogenic processes and the tumor microenvironment (232). Mutations in both *KDM6A/6B* have been identified, and copy number loss of these KDM6 subfamily members, predominantly in group 4 MB (54, 183). Inhibition of the histone demethylases KDM6A and KDM6B by a pharmacological inhibitor, GSKJ4, increased global H3K27me3 in K27M-mutant brainstem gliomas, leading to reduced tumor growth, improved survival, and sensitized tumor cells to radiation therapy in preclinical models (233). Other KDMs that have been shown to have altered expression in MB include KDM3A (H3K9me2/me1 histone demethylase), KDM4C (H3K36me2/me3 histone demethylase), KDM5A/B (H3K4me2/me3 histone demethylases), and KDM7A (dual H3K9 and H3K27 histone demethylase) (54, 56).

Group 3 MB has the highest potential for metastasis (55). MYC amplification is considered one of the defining features of group 3 MB (54, 234), although it is thought not to be sufficient to promote tumorigenesis alone (235, 236). More recently, other pathways promoting metastasis were identified independent of MYC amplification. Prune exopolyphosphatase 1 (Prune-1) enhances the TGF-β pathway, with subsequent upregulation of *Otx2* and *Snail* and downregulation of *Pten* (237). High expression of *Prune-1* and *Lsd1/Kdm1a* are reported in group 3 MB, and Bibbò et al. suggest that LSD1/KDM1A is an epigenetic regulator of *Prune-1* (238). *In vitro*, dual inhibition of PRUNE-1 and LSD1/KDM1A promoted differentiation and altered the tumor microenvironment in MB cells, identifying a potential therapeutic approach for group 3 MB tumors (238). Additionally, GFI1 and GFI1B work cooperatively with MYC to drive tumorigenesis in a subset of group 3 MB (239). Lee et al. (2019) demonstrated that Lsd1 physically interacts with Gfi1, and together, they are involved in the inhibition of genes involved in neuronal commitment and differentiation (240). The pharmacological inhibition of Lsd1 in Gfi1-driven MB *in vitro* and *in vivo* inhibits tumor cell growth and supports the idea that targeting Lsd1 may be an effective strategy for these tumors (240).

In cerebellar granular progenitor cells (CGPCs), the repressive PRC2 and G9A/G9A-like protein oppose the elevation of MLL4 and KDM7A activities to maintain REST homeostasis. However, alterations, either up- or downregulation in KDM7A activities, lead to dysregulation of REST homeostasis. Altered KDM7A expression in human SHH-MB leads to poor survival (241). This suggests that KDM7A may not act solely as a repressor or activator but may depend on the cellular context; therefore, targeting KDM7A in MB may require a nuanced approach to avoid unintended REST deregulation. This intricate interplay between KDM7A and REST homeostasis in SHH-MB exemplifies the challenge of targeting epigenetic regulators, such as KDMs and KMTs. The potential for unintended, system-wide effects due to the interconnected nature of epigenetic modifications highlights the necessity for comprehensive investigations to understand and mitigate these risks. This complexity extends beyond individual regulators. Disruption of global methylation patterns, or mutations in histone methyltransferases or demethylases, can cause alterations to normal cellular processes, contributing to oncogenesis by promoting uncontrolled cell growth and survival. Continued expansion of our understanding of the mechanisms regulating histone methylation in pediatric brain tumors, including the context-dependent roles of enzymes that regulate histone methylation will continue to provide insights into the biology of these cancers and help delineate potential and better treatment options for these devastating tumors.

#### 5.2.2 Histone acetylation

Histone acetylation, like methylation, was one of the first histone PTMs described, and it is a dynamic process governed by the opposing actions of histone acetyl transferases (HATs) and histone deacetylases (HDACs) (47). The balance between acetylation and deacetylation is crucial for regulating various cellular processes, including gene expression, DNA repair, and cell cycle progression. Dysregulation of HATs and HDACs can lead to aberrant acetylation patterns.

##### 5.2.2.1 Histone acetyl transferases

HATs add acetyl groups to lysine residues on histones lysine residues and are typically associated with open chromatin structure and enhanced gene expression (242). HATs serve as multifunctional transcriptional coactivators and facilitate acetylation of several histone lysine residues including H3K27 (243–246). H3K27ac is an activating mark associated with enhancers, super enhancers, and promoters. Pediatric brain tumors including MB, AT/RT, H3K27-altered DMG, H3-G34 DHG and ependymomas have distinct H3K27ac profiles that relate with epigenetic and tumor cell states (45, 66, 133, 148, 160, 161, 168, 176, 239, 247–253). HATs are classified into type A and B HATs. Type A HATs are subdivided into five families: the GNAT family, p300/CBP family, MYST family, basal TF family, and NRCF family (254). These proteins contain several homologous domains including the catalytic HAT domain and bromodomains (BRD) (243–246). In MB, somatic mutations involving the HATs *CREBBP* and *EP300* (encoding CBP/KAT3A and P300, respectively) affecting histone acetylation regulation are observed across all subgroups (54, 56, 73, 182, 184). Germline mutations in *CREBBP* are associated with Rubinstein-Taybi syndrome (RTS), a neurodevelopmental disorder that predisposes individuals to CNS malignancies, including MB (255–257). Interestingly, during embryonic development, the loss of *Crebbp* in GNPs impairs cerebellar development, whereas postnatal loss of *Crebbp* synergizes with SHH signaling to enhance the growth of MB (258). Schoof et al. found that concurrent loss of *CREBBP* function and MYCN overexpression in neural stem cells resulted in the development of aggressive forebrain tumors, suggesting a critical mechanistic link between these two factors in oncogenesis (259). Several cancer cell lines, including MB subtypes, are sensitive to CBP/EP300 inhibitors. Shendy et al. (2024) demonstrated that A485 (HAT domain inhibitor) and CCS1477 (BRD domain inhibitor) have varying effects across tumor types, with Group 3 MB exhibiting particular sensitivity to BRD inhibition (260). Together, these findings underscore the importance of understanding the complex roles of HATs in pediatric brain cancers, as understanding their specific contributions to oncogenesis may reveal useful therapeutic vulnerabilities.

Dysregulation of other HATs are reported in MB. For example, the downregulation of the histone H4 lysine K16-specific acetyltransferase (MOF) and lower H4K16 acetylation in MB has been associated with lower survival rates (261, 262). H4K16ac is associated with DNA damage repair and gene expression (263). Although not explicitly reported for MB, the loss of global H4K16ac in concert with MOF may lead to pathogenesis via dysregulation of oncogenes or tumor suppressor genes, increased genomic instability, or dysregulation of cell cycle (263–265).

##### 5.2.2.2 Histone deacetylases

In contrast to HATs, HDACs remove acetyl groups, which generally results in chromatin condensation and transcriptional repression (266). There are ~18 human HDACs, classified into five classes based on their sequence similarity to yeast deacetylases, domain composition, and dependence on specific cofactors (267). Alterations in HDAC expression have frequently been reported in pediatric brain cancers. In MB, increased HDAC2 expression is observed, especially in MYC-driven MB tumors (268, 269). Also, increased HDAC5 and HDAC9 expression is seen in a subset of MB and is associated with poor prognosis (270). In AT/RT, HDAC1 was reported to be differentially expressed, suggesting that HDAC inhibitors targeting HDAC1 may be beneficial compared to those with less specificity in young patients with ATRT (271).

Trichostatin A (TSA), the first reported potent and specific inhibitor of HDAC, led to alterations in cell proliferation and differentiation *in vivo* (272). Subsequently, vorinostat [also known as suberoylanilide hydroxamic acid (SAHA)], a Class I and Class IIb HDAC inhibitor, was shown to induce differentiation, growth arrest, and apoptosis *in vitro* (273, 274). Vorinostat effectively induces cell death in MB cell lines, patient-derived primary tumor cultures, and xenograft models, with minimal prohibitive toxicity observed in fibroblasts and animal models (4). In MB cell lines, the class I HDAC, HDAC2, is overexpressed in poor-prognosis subtypes, including SHH, Group 3, and Group 4, with MYC-amplified Group 3 cells demonstrating increased sensitivity to HDAC inhibition (269). Specifically, HDAC inhibition reduced metabolic activity and increased cell death in the MYC-amplified MB cells (269). Separately, high-throughput screening identified panobinostat (LBH-589), a pan-HDAC inhibitor, as highly effective against MYC-amplified Group 3 MB, with treatment decreasing MYC expression and inhibiting cell growth (275). To further elucidate the mechanism of HDAC inhibition in MYC-amplified MB, Ecker et al. (2020) investigated the interaction between MYC and HDAC2 and reported that HDAC inhibition disrupts the MYC-HDAC2 complex in MYC-amplified medulloblastoma, leading to reduced chromatin binding of MYC. This results in the downregulation of MYC-activated genes and the upregulation of MYC-repressed genes, effectively reversing the MYC-dependent transcriptional program and providing a therapeutic strategy for these MYC-amplified MB tumors (276).

A phase 1 trial in pediatric patients showed that Vorinostat was well-tolerated (277). However, despite promising preclinical and early clinical data, Vorinostat has shown limited efficacy in pediatric brain tumor clinical trials, including in DMGs (278). Ongoing trials are investigating Vorinostat in combination with other therapies for pediatric solid or CNS tumors (e.g., NCT02420613, NCT04308330, NCT06693284). Similarly, Panobinostat, another pan-HDAC inhibitor, suppressed MB leptomeningeal seeding in preclinical mouse models (279). Panobinostat was identified from a drug screening study to effectively kill H3K27M DMG cells (280). Panobinostat has been studied in several preclinical DMG models as monotherapy or in combination with various drugs (281–287). However, clinical trials of Panobinostat in DMGs have shown limited efficacy, although the drug was tolerated with expected toxicities, such as myelosuppression and diarrhea (288). Current Panobinostat trials are exploring blood-brain barrier disruption with focused ultrasound or direct intraventricular administration in DMG (NCT04804709, NCT04315064). Despite encouraging preclinical results and safety profiles, HDAC inhibitors have not demonstrated significant efficacy in pediatric brain tumors as monotherapy. Current strategies focus on combining HDAC inhibitors with cytotoxic chemotherapy, hypomethylating agents, or immunotherapy and on improving drug delivery across the blood-brain barrier. These approaches will be crucial for future clinical investigations. Overall, the role of HATs and HDACs in pediatric brain cancers highlights the therapeutic potential of both HAT and HDAC inhibitors.

## 6 Conclusion

The intricate landscape of pediatric brain cancers reveals a critical reliance on precise chromatin regulation and histone post-translational modifications. Unlike many adult tumors, pediatric malignancies do not bear a high burden of recurrent mutations, but rather aberrant epigenetic modifications. These disruptions, encompassing alterations in histone proteins, PTMs, and chromatin remodeling complexes, profoundly influence gene expression and contribute to oncogenesis. The diverse roles of histone modifications and the enzymes that regulate them—writers, readers, and erasers—underscore the complexity and importance of this field. By focusing on the impact of chromatin biology and modifications in pediatric brain tumors, we gain crucial insights into disease mechanisms and identify potential therapeutic targets for correcting aberrant chromatin states. Ultimately, a deeper understanding of these epigenetic vulnerabilities will pave the way for developing more effective and targeted therapies for these devastating childhood cancers.
